# Comparison of the predictive ability of clinical frailty scale and hospital frailty risk score to determine long-term survival in critically ill patients: a multicentre retrospective cohort study

**DOI:** 10.1186/s13054-022-03987-1

**Published:** 2022-05-03

**Authors:** Ashwin Subramaniam, Ryo Ueno, Ravindranath Tiruvoipati, Velandai Srikanth, Michael Bailey, David Pilcher

**Affiliations:** 1 Department of Intensive Care, Frankston Hospital, Peninsula Health, 2 Hastings Road, VIC 3199 Frankston, Australia; 2 Australian and New Zealand Intensive Care Research Centre, Department of Epidemiology and Preventive Medicine, Monash University, Melbourne, VIC Australia; 3 Peninsula Clinical School, Monash University, Frankston, VIC Australia; 4 Department of Intensive Care, Eastern Health, Box Hill, VIC Australia; 5 Department of Geriatric Medicine, Peninsula Health, Frankston, VIC Australia; 6 National Centre for Healthy Ageing, Melbourne, Australia; 7 Centre for Outcome and Resource Evaluation, Australian and New Zealand Intensive Care Society, Melbourne, VIC Australia; 8 Department of Intensive Care, Alfred Hospital, Melbourne, VIC Australia

**Keywords:** Frailty, Clinical frailty scale, Hospital frailty risk score, CFS, HFRS, Long-term outcomes, 1-year survival

## Abstract

**Background:**

The Clinical Frailty Scale (CFS) is the most commonly used frailty measure in intensive care unit (ICU) patients. The hospital frailty risk score (HFRS) was recently proposed for the quantification of frailty. We aimed to compare the HFRS with the CFS in critically ill patients in predicting long-term survival up to one year following ICU admission.

**Methods:**

In this retrospective multicentre cohort study from 16 public ICUs in the state of Victoria, Australia between 1st January 2017 and 30th June 2018, ICU admission episodes listed in the Australian and New Zealand Intensive Care Society Adult Patient Database registry with a documented CFS, which had been linked with the Victorian Admitted Episode Dataset and the Victorian Death Index were examined. The HFRS was calculated for each patient using the International Statistical Classification of Diseases and Related Health Problems, Tenth Revision (ICD-10) codes that represented pre-existing conditions at the time of index hospital admission. Descriptive methods, Cox proportional hazards and area under the receiver operating characteristic (AUROC) were used to investigate the association between each frailty score and long-term survival up to 1 year, after adjusting for confounders including sex and baseline severity of illness on admission to ICU (Australia New Zealand risk-of-death, ANZROD).

**Results:**

7001 ICU patients with both frailty measures were analysed. The overall median (IQR) age was 63.7 (49.1–74.0) years; 59.5% (*n* = 4166) were male; the median (IQR) APACHE II score 14 (10–20). Almost half (46.7%, *n* = 3266) were mechanically ventilated. The hospital mortality was 9.5% (*n* = 642) and 1-year mortality was 14.4% (*n* = 1005). HFRS correlated weakly with CFS (Spearman’s rho 0.13 (95% CI 0.10–0.15) and had a poor agreement (kappa = 0.12, 95% CI 0.10–0.15). Both frailty measures predicted 1-year survival after adjusting for confounders, CFS (HR 1.26, 95% CI 1.21–1.31) and HFRS (HR 1.08, 95% CI 1.02–1.15). The CFS had better discrimination of 1-year mortality than HFRS (AUROC 0.66 vs 0.63 *p* < 0.0001).

**Conclusion:**

Both HFRS and CFS independently predicted up to 1-year survival following an ICU admission with moderate discrimination. The CFS was a better predictor of 1-year survival than the HFRS.

**Supplementary Information:**

The online version contains supplementary material available at 10.1186/s13054-022-03987-1.

## Background

Clinical frailty describes a state of decline in physical, physiologic and cognitive reserve [1]. Frailty increases with age and is characterised by poor mobility, weakness, reduced muscle mass, poor nutritional status and diminished cognitive function.^1^ Frailty has been associated with falls, prolonged hospitalisation, and delayed recovery from illness and surgery. Frail individuals require more support with activities of daily living and are more susceptible to adverse events and death when compared to age-stratified non-frail individuals [2,3]. Frailty, across the adult age spectrum, is common in patients that are admitted to intensive care units (ICU) [2,4]. Patients with frailty generally have poorer hospital and 1-year outcomes, poorer quality of life, disability and functional dependence ^[[[[[2–6]]]]]^.

In ICU, frailty has been most commonly assessed [3,7,8] using the clinical frailty scale (CFS) [9], and the frailty index [10]. The ease of use, with clinical descriptors and pictographs, has made the CFS the most commonly used frailty measure [9]. Despite having some limitations, including subjectivity in assessment and using a judgement-based score, the CFS is validated to stratify older adults according to the level of vulnerability [9] and reliably predict poor short and longer-term outcomes in critically ill patients [4,5,11,12].

Hospital frailty risk score (HFRS) is a novel administrative frailty measure to identify patients at risk of frailty [13]. This is a validated tool to assess frailty in hospitalised patients [14]. HFRS is based on the International Statistical Classification of Diseases and Related Health Problems, Tenth Revision (ICD-10) coding system obtained from the Australian-refined Diagnosis-related groups (AR-DRG). The resulting score is categorised as low-risk (< 5), intermediate-risk (5–15) and high-risk (> 15). Patients with intermediate-risk and high-risk categories were classified as being frail [13]. However, there is conflicting evidence when it comes to the validity in ICU patients, especially in older patients [15–17].

A frailty tool that can predict long-term survival and other clinically relevant outcomes is more likely to be valid in assessing at-risk patients with frailty. The primary aim of the study was to compare the HFRS with the CFS in critically ill patients in predicting long-term survival up to 1 year following ICU admission.

## Methods

### Ethics approval

This study was approved by The Research Governance of Peninsula Health Ethics Committee (reference number HREC/47502/PH-2018, DHHS/RQ907) with a waiver of informed consent.

### Study design, setting and patients

 We conducted a retrospective multicentre observational study from 1st April 2017 to 30th June 2018 including consecutive critically ill patients admitted to 16 public ICUs in the state of Victoria, Australia with a clinical documented CFS score. The censor date for survival follow-up was 31st July 2018 to ensure that there was at least one-month follow up for all patients. We only included the first hospital admission during the study period.

### Data sources and measurement

*Australia and New Zealand Intensive Care Society (ANZICS) Adult Patient Database (APD)* This bi-national clinical quality registry dataset collects de-identified information on all admissions to contributing adult ICUs in Australia and New Zealand. Trained staff working in each ICU collect this data. All ICUs within public hospitals in Victoria contributed throughout the study period. Apart from each patient’s demographic details, the data also captured their diagnostic, biochemical, physiological, and chronic health parameters from the first 24 h of ICU admission as required to calculate illness severity scores. The definitions are described in the ANZICS-APD data dictionary [18].

*Victorian Admitted Episodes Dataset (VAED)* All Victorian public hospitals submit data to the Victorian Department of Health and Human Services. This administrative dataset contains the ICD-10 coded diagnostic information, demographic data, and outcomes for all hospitalisations.

*Victorian Death Index (VDI)* This administrative dataset records the date and cause of all deaths that occur in Victoria, based on the issued death certificates. Any deaths that occur outside Victoria are not included in the registry. Information was available up to 31^st^ July 2018.

Probabilistic methods were used to match de-identified ICU admission episodes listed in the ANZICS registry to their equivalent administrative data by the Centre for Victorian Data Linkage.

### Definitions of Frailty

The CFS is voluntarily collected as part of the ANZICS-APD at the time of ICU admission, based on the patient’s level of physical function in the two months before ICU admission by sixteen out of twenty-three hospitals. The CFS (range 1–8) categorises patients as non-frail (1 = very fit; 2 = well; 3 = managing well; 4 = vulnerable) or frail (5 = mildly frail; 6 = moderately frail; 7 = severely frail; 8 = very severely frail). Patients with a score ≥ 5 were considered frail. CFS was assigned by data collectors in participating ICUs based on the patient’s level of physical function in the two months preceding ICU admission [18].

The HFRS (range 0 to 45) was estimated using routine data based on the ICD-10 coding system obtained from the AR-DRG [13,19]. ICD-10 codes used to estimate HFRS, and the respective points awarded for each diagnosis are summarised in Additional file 1: Table S1. For this study, we categorised patients with HFRS score > 5 as frail and those with score < 5 as non-frail [13]. We only used the ICD-10 codes that represented pre-existing conditions at the time of index hospital admission, rather than those that were developed during the hospitalisation. A complete list of ICD-10 variables extracted from the VAED database is listed in Additional file 1: Table S2. VDI database is summarised in Additional file 1: Table S3. The patients were categorised as non-frail and frail for both CFS and HFRS.

### Study aims and outcomes

The primary aim was to assess the use of HFRS as a frailty screening tool in ICU patients by comparing its performance with the CFS as a predictor of one-year survival following ICU admission. The secondary aims were to compare the performance of the CFS and the HFRS as predictors of ICU, hospital, 28-day, 90-day, 6-month and 1-year mortality. The pre-defined subgroup analyses included prediction of 1-year mortality for patients ≥ 75 years of age and those needing mechanical ventilation.

### Statistical analysis

Categorical comparisons between frail and non-frail patients were performed using Chi-square, two-sample *t*-tests for normally distributed data and Wilcoxon rank-sum test otherwise, with results reported as counts (%), means [standard deviation (SD)] or median (interquartile range [IQR]), respectively. Correlation between the continuous CFS and HFRS was assessed using Spearman correlation coefficient and agreement using Kappa for dichotomous (not frail: CFS 1–4, HFRS 0–5; frail: CFS ≥ 5, HFRS > 5). Patient survival was compared using Cox proportional hazards regression adjusting for patient’s illness severity, and sex, with results reported as hazard ratios (HR, 95% CI). Time-dependent covariate analysis and log-minus-log plots were performed to assess proportionality assumptions. While HR were reported as the risk associated with a 1-unit increment for the CFS (range 1–8), to facilitate a more proportional comparison between the 2 frailty tools, HR for HFRS were reported as the risk associated with a 5-unit increase (range 0–45). The dichotomised Kaplan–Meier survival curves were performed for the two frailty measures. The performance of the CFS and the HFRS in predicting time-specific mortality rates was determined using logistic regression models with results reported as the area under the receiver operating characteristic (AUROC) plots with comparison using chi-square tests [20]. Multivariable logistic regression analysis was used to compare the performance of the CFS and the HFRS as predictors of ICU, hospital, 28-day, 90-day, 6-month and 1-year mortality. Illness severity was determined using the Australian and New Zealand Risk of death (ANZROD) which is a highly predictive mortality prediction model used for benchmarking ICU performance in Australia and New Zealand. ANZROD includes components of the APACHE III/IV scoring system, such as age, chronic illnesses, acute physiological disturbance and diagnosis, and the presence of treatment limitation on admission to ICU and provides an accurate estimate of the severity of illness in the first 24 h of ICU admission [21,22]. Hence, adjusting for sex was required as a separate variable, and not age. Additional sensitivity analyses were performed based on elective and non-elective admissions and those who were discharged home alive. The HFRS was quantified with the ICD-10 codes from the linked dataset using R software, version 3.5.0 (The R Foundation). The data analysis was performed using SPSS Version 27 (IBM). A two-sided p value of 0.05 was used to indicate statistical significance.

## Results

During the study period, there were a total of 20,457 hospitalisations from 16 hospitals (9 rural, 3 metropolitan and 4 tertiary) listed in the ANZICS adult patient database. Of these, 14,943 (73%) were linked with the VAED and VDI datasets. Of the linked admissions, there were 7451 patients with a documented CFS, the ICD-10 codes were available to estimate HFRS. 450 patients who were readmitted to the ICU during the same hospital stay were excluded. The final study dataset comprised 7001 patients from whom both the CFS and HFRS measures were available. Additional file 1: Table S4 illustrates the comparison between included (*n* = 7001) vs excluded (*n* = 13,457). While there are some differences between groups, there was no difference for hospital or ICU mortality, ICU length of stay or APACHE 3.

The overall median (IQR) age was 63.7 (49.1–74.0) years; 59.5% (*n* = 4166) were male. Overall, 2390 patients (34.1%) were ≥ 70 years of age and 35.5% of these (848/2390) were ≥ 80 years of age. The prevalence estimates of frailty measured by CFS and HFRS across different age categories are provided in Additional file 1: Figure S1. The demographic characteristics, illness severity scores and proportion requiring mechanical ventilation are presented in Table 1.

**Table 1:** Demographics, physiological, illness severity, comorbidities, and outcomes among critically ill patients

	All (*N* = 7001)	Clinical frailty scale (CFS)^(1)^		Hospital frailty risk score (HFRS)^(2)^	
		Non-frail (< 5) (*n* = 5678, 81.1%)	Frail (≥ 5) (*n* = 1323, 19.9%)	Non-frail (< 5) (*n* = 5164, 73.8%)	Frail (≥ 5) (*n* = 1837, 26.2%)
Demographics					
Age, median (IQR)	63.7 (49.1–74.0)	61.8 (47.0–72.2)	69.9 (59–1-79.6)	63.3 (49.2–73.1)	64.6 (48.9–76.2)
Male	59.5 (4166)	61.0 (4590)	53.0 (1013)	59 (3048)	60.9 (1118)
Type of hospital, % (*n*)					
Metropolitan	11.6 (812)	10.4 (588)	16.9 (224)	9.9 (511)	16.4 (301)
Rural/Regional	26.6 (1863)	26.0 (1474)	29.4 (389)	29.1 (1505)	19.5 (358)
Tertiary	61.8 (4326)	63.7 (3616)	53.7 (710)	61.0 (3148)	64.1 (1178)
Admission source, % (*n*)					
Private residence	77.1 (5397)	76.9 (4367)	77.8 (1030)	78.4 (4050)	(1347)
Transfer from RACF	22.1 (1545)	22.6 (1283)	19.8 (262)	21.0 (1084)	(461)
Transfer from rehabilitation	0.3 (22)	0.1 (8)	1.1 (14)	0.2 (10)	(12)
No information	0.5 (35)	0.4 (20)	1.1 (15)	0.4 (19)	0.9 (16)
Admission type to ICU, % (*n*)					
Elective surgery	24.9 (1740)	27.0 (1533)	15.6 (207)	32.3 (1670)	(70)
Emergency surgery	20.2 (1415)	19.9 (1129)	21.6 (286)	20.9 (1077)	(338)
Medical Admission	54.9 (3846)	53.1 (3016)	62.7 (830)	46.8 (2417)	77.8 (1429)
Comorbidities, % (*n*)					
Chronic respiratory disorders	7.7 (539)	5.0 (282)	19.4 (257)	8.5 (439)	(100)
Chronic cardiovascular disorders	6.7 (470)	5.1 (288)	13.8 (182)	6.8 (349)	(121)
Chronic renal failure	3.7 (260)	2.9 (162)	7.4 (98)	2.9 (152)	(108)
Immune disorder	2.2 (151)	1.5 (86)	4.9 (65)	2.1 (110)	(41)
Immunosuppressive disorder	5.6 (395)	4.6 (261)	10.1 (134)	5.3 (272)	(123)
Cirrhosis / Hepatic failure	2.6 (181)	2.3 (129)	3.9 (52)	2.3 (121)	(60)
Metastatic cancer	2.6 (183)	2.1 (121)	4.7 (62)	2.7 (141)	(42)
Leukaemia	1.6 (111)	1.4 (81)	2.3 (30)	1.4 (70)	(41)
Lymphoma	0.7 (52)	0.7 (37)	1.1 (30)	0.8 (39)	0.7 (13)
Pre-ICU hours, hours, median (IQR)	43.9 (135.9)	7.2 (3.8–18.2)	9.0 (4.3–27.5)	8.2 (4.2–24.0)	5.7 (3.2–12.7)
Treatment limitation, % (n)	9.6 (667)	6.1 (343)	24.5 (324)	7.6 (384)	15.0 (275)
ICU Admission post MET call, % (n)	12.7 (885)	11.0 (625)	19.7 (260)	11.5 (591)	16.0 (294)
Illness severity Scores, median (IQR)					
APACHE II score	15.4 (7.5)	14 (9–19)	18 (14–23)	13 (10–18)	18 (13–23)
APACHE III score	51.9. (24.7)	46 (33–62)	58 (45–74)	46 (33–60)	58 (42–76)
ANZROD (%) mean (SD)	9.4 (17)	8.1 (16)	15.2 (19.6)	7.3 (15)	15 (20)
Charlson comorbidity index	0 (0–2)	0 (0–1)	0 (0–2)	0 (0–1)	1 (1–2)
Outcomes, % (*n*)					
ICU mortality	6.7 (472)	5.8 (328)	10.9 (144)	5.5 (286)	(186)
Hospital mortality	9.2 (642)	7.6 (433)	15.8 (209)	7.5 (385)	(257)
28-day mortality	9.0 (630)	7.4 (420)	15.9 (210)	7.3 (378)	(252)
90-day mortality	11.8 (828)	9.4 (533)	22.3 (295)	9.5 (493)	(335)
6-month mortality	13.5 (944)	10.6 (600)	26.0 (344)	11.1 (571)	(373)
12-month mortality	14.4 (1005)	11.3 (642)	27.4 (363)	11.8 (609)	(396)
ICU LOS, hours, median (IQR)	44.8 (23.1–87.7)	42.9 (22.4–83.7)	55.8 (29.3–100.6)	40.9 (21.8–72.7)	64.7 (34.7–125.6)
Hospital LOS, days, median (IQR)	8 (4–15)	7 (4–14)	9 (5–8)	7 (4–14)	10 (5–20)
Organ failure and supports, % (*n*)					
Mechanical ventilation	46.7 (3266)	47.9 (2722)	41.1 (544)	46.7 (2414)	(852)
Renal replacement therapy	6.8 (474)	6.1 (348)	9.5 (126)	5.2 (266)	11.3 (208)
Discharge destination, % (*n*)					
Hospital mortality	642 (9.2)	433 (7.6)	209 (15.8)	385 (7.5)	257 (4.0)
Usual residence	4515 (64.5)	3895 (68.6)	620 (46.9)	3723 (72.1)	792 (3.1)
Rehabilitation	932 (13.3)	671 (11.8)	261 (19.7)	458 (8.9)	474 (5.8)
New nursing home	110 (1.6)	43 (0.8)	67 (5.1)	69 (1.3)	41 (2.2)
Other^^^	801 (11.4)	636 (11.2)	165 (12.5)	528 (10.2)	273 (14.9)

*SD* standard deviation, *SOFA* Sequential organ failure assessment, *SAPS 3* Simplified Acute Physiology admission score, *n* number, *IQR* interquartile range, *ICU* intensive care unit, *LOS* length of stay, *RACF* Residential aged care facility, *TCP* transitional care program, *COPD* chronic obstructive pulmonary disease, *NYHA* New York Heart Association, *ICU* intensive care unit, *ANZROD* ANZ Risk of death score, *APACHE* Acute Physiology And Chronic Health Evaluation, *RoD* risk of death

Respiratory disorders: Chronic restrictive, obstructive disease resulting in severe exercise restriction (unable to climb stairs or perform household duties); or documented chronic hypoxia, hypercapnia, secondary polycythaemia, severe pulmonary hypertension (mean > 40 mmHg); or ventilator dependency

^(1)^Except for Admission source (*p* = 0.01), Leukaemia (*p* = 0.04) and Lymphoma (*p* = 0.08) all comparisons between frail and not-frail for CFS scores were statistically significant (*p* < 0.0001)

^(2)^With the exception of Age (*p* = 0.60), gender (*p* = 0.17) Chronic Cardiovascular (*p* = 0.80), Immune disorder (*p* = 0.80), Immunosuppression (*p* = 0.02), Cirrhosis (*p* = 0.03), Metastatic cancer (*p* = 0.31), Leukaemia (*p* = 0.01), Lymphoma (*p* = 0.84) and Mechanical Ventilation (*p* = 0.79) all comparisons between frail and not-frail for HFRS scores were statistically significant (p < 0.0001)

^*^ Other = unknown (*n* = 24); other hospital, incl. ICU (*n* = 711); another ICU from same hospital (*n* = 37)

^ includes discharge to hospital in the home (*n* = 19, 0.3%), mental residential care facility (*n* = 73, 1%), other (*n* = 38, 0.5%), left against medical advice (*n* = 155, 2.2%), statistical separation (*n* = 255 3.6%)

**Cardiovascular:** New York Heart Association Class IV: angina or symptoms at rest or on minimal exertion (whilst getting dressed or during self-care)

**Liver: *Biopsy proven*** cirrhosis and portal hypertension, or episodes of past upper GI bleeding attributed to portal hypertension. If the patient has a functioning liver transplant, this chronic health item does not apply

**Renal:** Must be receiving chronic haemodialysis or peritoneal dialysis

**Immune Suppressive Disease (Immune disease):** The patient has a disease that is sufficiently advanced to suppress resistance to infection: leukaemia, AIDS, lymphoma, severe autoimmune disease or documented diffuse metastatic cancer

**Immunosuppressive Therapy (Immunosuppressed):** The patient has received therapy that has suppressed resistance to infection: e.g. immunosuppression, chemotherapy within 4 weeks of admission, radiation, high-dose steroid treatment (e.g. > 1.5 mg/kg methylprednisolone or equivalent for ≥ 5 days), long term treatment with > 20 mg/day steroid

*Comparison between CFS and HFRS* The number of patients quantified as frail differed between the two frailty scores. Compared to the CFS, a higher proportion of patients were categorised as frail by the HFRS (18.9% for CFS [*n* = 1323] vs. 26.2% for HFRS [*n* = 1837] (Table 1). The overall hospital mortality was 9.2% (*n* = 642). The HFRS weakly correlated with the CFS (Spearman’s rho = 0.13 [95% CI 0.10–0.15]; *p* < 0.0001) (Table 2) and had poor agreement (kappa = 0.12 [95% CI 0.10–0.15]; *p* < 0.0001).

**Table 2:** Spearman correlation and Kappa agreement between the two frailty measures

HFRS	CFS score		Spearman's Correlation^(1)^	Agreement^(2)^
	Non-frail (CFS < 5)	Frail (CFS ≥ 5)	Correlation coefficient (95% CI)	Kappa
All patients (*n* = 7001)	5678	1323		
HFRS (non-frail; *n* = 5164 [73.8%])	4332	832	0.13 (0.10–0.15)	0.12 (0.10–0.15)
HFRS (frail; *n* = 1837 [26.2%])	1346	491		

*HFRS* hospital frailty risk score, *CFS* Clinical Frailty Scale

^(1)^ Spearman correlation based on continuous variables (*p* value was < 0.001)

^(2)^ Kappa agreement based on dichotomous variables (Except for Kappa agreement for patients needing mechanical ventilation (*p* = 0.004), all others had a *p* value of < 0.001)

**Fig. 1:**
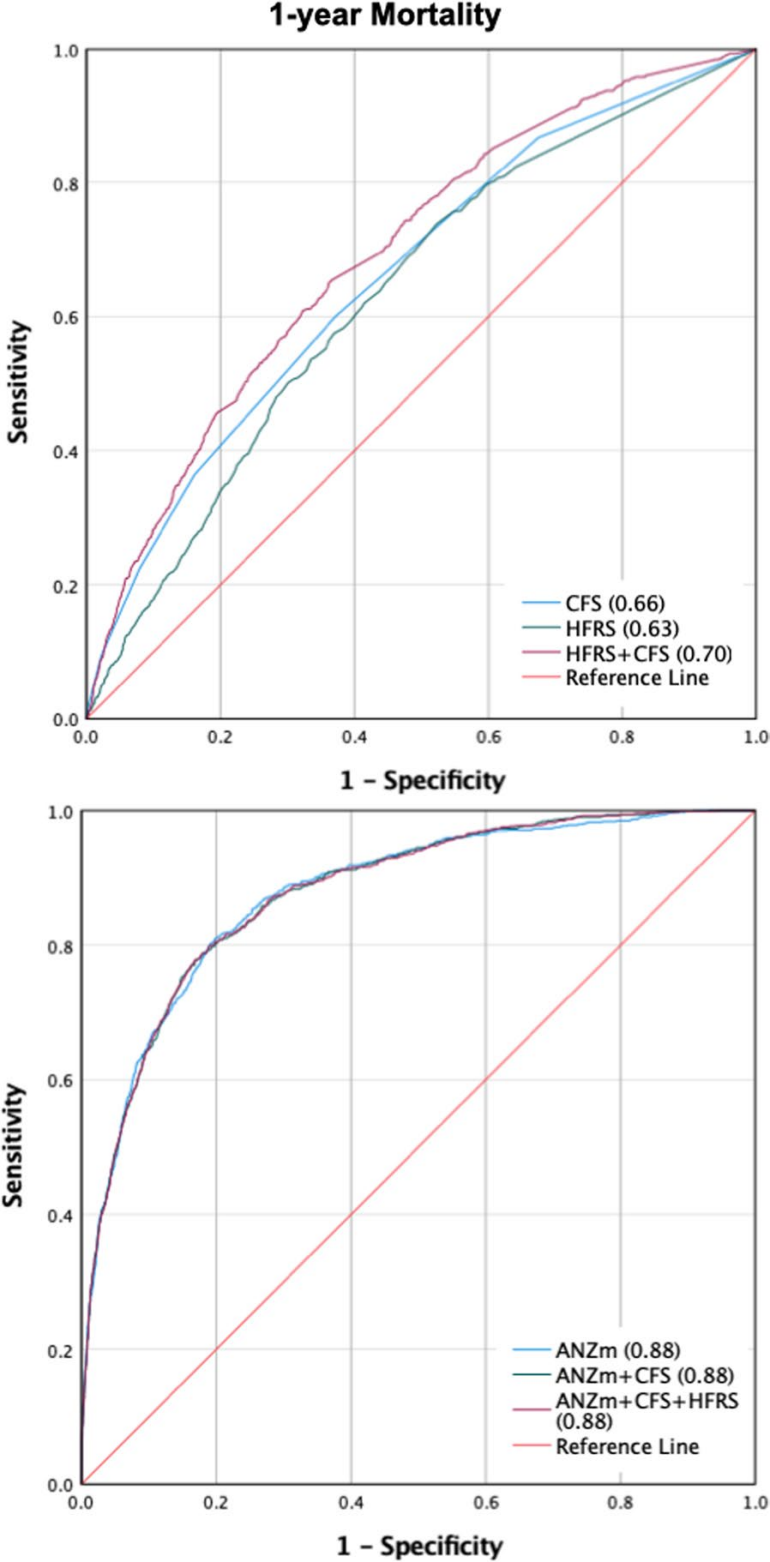
Area under the receiver operator curve for 1-year mortality

*Primary outcome: long-term survival* The categorical (frail vs. non-frail) unadjusted 1-year mortality rates were similar for the two frailty measures. The Cox proportional hazards regression (both CFS and HFRS as ordinal variables), adjusted for ANZROD, and sex, showed that both the CFS (1-unit increment; HR 1.26, 95% CI 1.21–1.31) and HFRS (5-unit increment; HR 1.08, 95% CI 1.02–1.15) were associated with long-term survival up to one year (Table 3). The categorised Kaplan–Meier survival curves for the two frailty measures demonstrated greater survival separation between non-frail and frail patients for CFS than HFRS (Additional file 1: Figure S2). When considering unadjusted models, CFS had significantly better discrimination than HFRS (AUROC 0.66 vs 0.63 *p* < 0.0001) for 1-year mortality. When CFS and HFRS were combined into a single model, the resulting AUROC further increased to 0.70 suggesting that both frailty measures were capturing unique variation in frailty outcome. However, after multivariable adjustment, neither frailty measurement was able to improve on the discrimination provided by patient illness severity assessed by ANZROD (Fig. 1). Additional file 1: Figure S3 illustrates the AUROC performance for CFS and HFRS for other mortalities.

**Table 3:** Unadjusted and adjusted Cox proportional hazards regression for CFS and the HFRS as continuous variables, adjusting for ANZROD, and sex CFS and HFRS for all patients

	Unadjusted	Adjusted*
	HR (95% CI)	HR (95% CI)
CFS	1.43 (1.37–1.48)	1.26 (1.21–1.31)
Male sex	–	1.11 (0.98–1.26)
ANZROD	–	1.05 (1.04–1.05)
HFRS**	1.38 (1.30–1.45)	1.08 (1.02–1.15)
Male sex	–	1.03 (0.90–1.17)
ANZROD	–	1.05 (1.05–1.05)

*Adjusted for sex and ANZROD

**5-unit increase HFRS was used to calculate the HR

*CFS* Clinical frailty score, *HFRS* hospital frailty risk score, *HR* Hazard ratio, *ANZROD* Australia and New Zealand risk of death

**Fig. 2:**
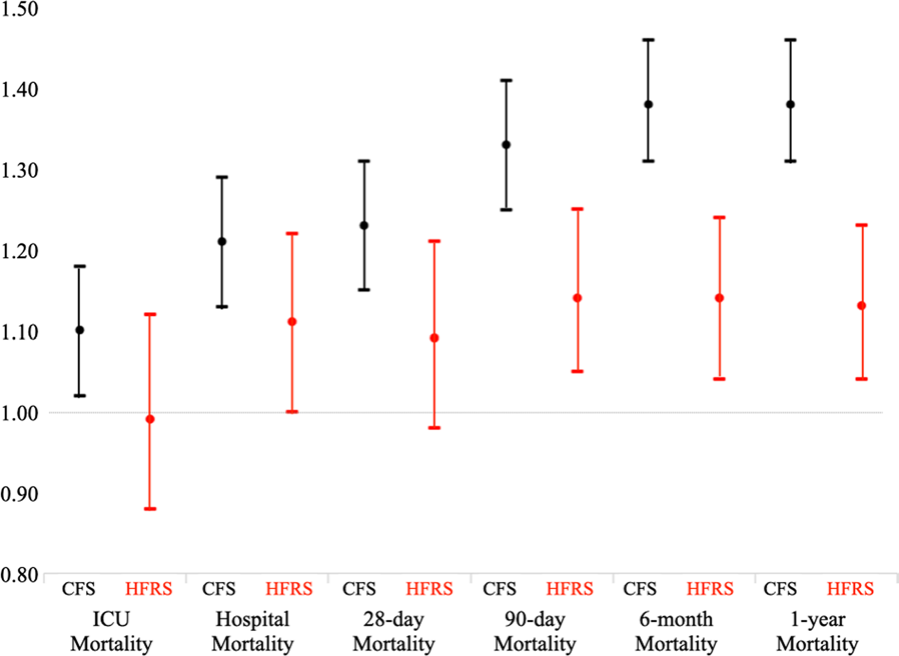
Odds ratio for short- and long-term mortality between CFS and HFRS treated as a dichotomous variable for all patients

*Secondary outcomes* The multivariable logistic regression, adjusted for ANZROD and sex, demonstrated that the CFS independently predicted mortality at hospital discharge, 28-day, 90-day, 6-month, and 1-year for frail patients. The HFRS was independently predictive of only 90-day and 1-year mortalities (Additional file 1: Table S5, Fig. 2), but the magnitude of prediction was lower than the CFS prediction.

### Subgroup analysis

*Patients* ≥ *75 years of age* There were 1,683 patients 75 years and over. The HFRS weakly correlated with the CFS (Spearman’s rho = 0.22 [95% CI 0.18–0.27]; *p* < 0.0001) and had a poor agreement (Kappa = 0.05; 95% CI 0.01–0.08; *p* = 0.004; Additional file 1: Table S5). Although the AUROC curves had moderate discrimination the scores were similar for both CFS and HFRS (Additional file 1: Figure S4). The HFRS (5-unit increment; HR 1.05, 95% CI 0.95–1.16), adjusted for ANZROD and sex, was not independently associated with 1-year survival for HFRS when compared with CFS (1-unit increment; HR 1.18, 95% CI 1.10–1.27; Additional file 1: Table S6). Although the HFRS was independently predictive of only 90-day, 6-month and 1-year mortalities (Additional file 1: Table S7), the magnitude of prediction was weaker than the CFS prediction.

*Patients needing mechanical ventilation* 3266 patients received mechanical ventilation. While the HFRS did not significantly correlate with the CFS (Spearman’s rho = 0.02 (95% CI − 0.05, 0.02; *p* = 0.40), the AUROC curves indicated moderate discrimination with CFS AUROC lower than HFRS (0.60 vs 0.63 p < 0.0001; Additional file 1: Figure S4). After adjustment, both CFS (1-unit increment; HR 1.14; 95% CI 1.08–1.20) and HFRS (5-unit increment; HR 1.17, 95% CI 1.08–1.28) were independently associated with survival up to one year (Additional file 1: Table S6). While the CFS was predictive of both short- and long-term mortalities, HFRS was not (Additional file 1: Table S7).

### Sensitivity analysis

*Elective versus non-elective admissions* 1740 patients were electively admitted, while 5261 were non-elective admissions. There were no differences in correlation, agreement, or 1-year survival based on their admission type (Additional file 1: Tables S5 and S6). The HFRS was independently predictive only for 90-day mortality for electively admitted patients, while the CFS was independently predictive only for 6-month and 1-year mortalities (Additional file 1: Table S7). Contrarily, the HFRS was not predictive of both short- and long-term mortalities for non-elective admissions, whereas the CFS was independently predictive of both short- and long-term mortalities (Additional file 1: Table S7).

*Outcomes of patients who survived to hospital discharge* Of the 6359 patients who were discharged home alive, 364 patients (5.7%) subsequently died (13.8% [154/1114] were frail and 4.0% [210/5245] were non-frail, based on CFS). The HFRS weakly correlated with the CFS (Spearman’s rho = 0.12 [95% CI 0.09–0.15]; *p* < 0.001) and had a poor agreement (Kappa = 0.12; 95% CI 0.07–0.14; *p* < 0.001; Additional file 1: Table S5). Both HFRS (5-unit increment; HR 1.13, 95% CI 1.02–1.25) and CFS (1-unit increment; HR 1.51, 95% CI 1.42–1.61), adjusted for ANZROD and sex, were independently associated with 1-year survival, but the magnitude of prediction for HFRS was weaker than the CFS prediction (Additional file 1: Table S6). Although the HFRS was independently predictive of only 90-day, 6-month and 1-year mortalities (Additional file 1: Table S7), the magnitude of prediction was weaker than the CFS prediction.

## Discussion

### Key findings

This large multicentre retrospective cohort study demonstrated both CFS and HFRS independently predicted 1-year survival with moderate discrimination. The CFS had better prediction than the HFRS. Both frailty measures were independently predictive and moderately discriminatory in differentiating long-term survivors from patients who died up to one year after ICU admission. The use of CFS to categorise patients as frail resulted in greater separation of Kaplan Meier curves. Neither CFS nor HFRS provided additional discriminatory value over and above that provided by the acute assessment of illness severity measured using ANZROD, however, the increased area under the curve for the combination of the two measures suggested they may both be measuring different aspects of frailty. Finally, frailty diagnosed using the CFS was predictive of both short- and long-term mortality in mechanically ventilated patients and those who were admitted non-electively, but HFRS was not.

### Relationship with previous studies

There is no gold standard for frailty measure in critically ill patients. Diagnostic confirmation of frailty requires a comprehensive assessment across a spectrum of contributing domains, which are impractical and challenging in ICU settings. The validated HFRS, on the contrary, is estimated using administrative data. Both CFS and HFRS are readily interpreted by non-geriatric specialists and hence well suited for screening at-risk patients with frailty [23]. However, there is controversy of the performance of these two frailty measures.

The lower prevalence of HFRS-categorized frailty in our study compares with the reported literature (58–67% in hospitalised patients [13,24], and 59% in an ICU cohort [25]). The higher published prevalence is likely because the HFRS heavily depends on simple counts of co-morbidities for frailty classification generally without being able to differentiate pre-existing conditions from those which develop or are identified de-novo during the hospitalisation. By only including the pre-existing ICD-10 codes to determine HFRS, we observed that the prevalence of frailty was comparable to CFS-categorized frailty (26.2% and 18.9%, respectively) in our study. This was also relative to the recent meta-analysis that pooled ten observational studies that estimated a frailty prevalence of 30% among patients in ICU settings.

Higher age, comorbidity burden and primary diagnosis have previously been related to decreased survival in all patient groups following ICU admission [26,27]. CFS has been validated to predict short-term and long-term mortality in critically ill patients [4,6,11,28–31]. HFRS however relies on patient records. ICD-10 codes cannot reflect disease severity; they are normally used for reimbursement purposes. We observed that the CFS had greater predictive validity of long-term survival than the HFRS, further strengthening the argument that the CFS is a better and more reliable frailty screening measure in critically ill patients. The CFS meets all the criteria: i.e., being multi-dimensional, time-efficient, accurate and also simple. While the HFRS might be useful to assess morbidity, however, frailty is more than just the sum of comorbidities. Although the HFRS has been shown to predict ICU readmission [25,32], it relies on patient records, which are prone to be incomplete or possibly incorrect. As previously observed by Flaatten and colleagues [2], our study found that it significantly fell short in evaluating the true burden of frailty.

The HFRS, created for hospitalised but not ICU patients, was originally developed as low-risk, medium-risk, and high-risk (0–5, 5–15, > 15) and not dichotomised. The ICD-10 codes were arbitrarily selected. Furthermore, HFRS uses weights (i.e., points for each diagnosis) based on the coefficients of likelihood ratios in UK cohorts; thus, it may not be appropriate to use exactly the same weight in Australian ICU populations. For instance, 2.3 points for "Other disorders of fluid, electrolyte and acid–base balance" (observed in 33% of our cohort). Similarly, 3.2 points for "Delirium, not induced by alcohol and other psychoactive substances" (observed in 12% of our cohort). Future studies should focus on re-calculating the weight for each diagnosis using a larger Australian cohort.

Previous evidence has exhibited the challenges in comparing results of different frailty scales, with a 2017 review of 35 different frailty scores demonstrating significantly different degrees of agreement between scales in a longitudinal study of over 5000 older community-dwelling participants [33] however, HFRS was not included in that review. Our study demonstrated poor agreements between the CFS and the HFRS. The most likely explanation is that both measures estimate frailty based on different concepts of frailty.

### Study implications

Although the HFRS correlated weakly with poor agreements and a lower magnitude of independently predicting longer-term survival than the CFS, both frailty measures have some validity and may be beneficial in different situations. These two frailty tools measure different aspects: the CFS is a clinical assessment, that is more likely to be immediately available to clinicians and does have some validity for differentiating long-term survivors from those who die, even before the calculation of acute illness severity. On the other hand, the HFRS is likely to be available to administrators and health departments since it is collected in and calculable from routine coding with the data. The CFS and ANZROD are less likely to be available than administrators and health departments. However, by using only the ICD-10 codes from only the pre-existing conditions, the frailty status could be extrapolated as being recorded at the time of the indexed hospitalisation. This implies that an automated HFRS could be made available when the patient is hospitalised again or readmitted to ICU. Furthermore, the combined HFRS and CFS AUROC model demonstrated that both measures could explain differing proportions of variation associated with outcome, so both would be useful if ANZROD was not available. Furthermore, as ANZROD requires 24 h of data whereas HFRS and CFS do not, a prediction model for death using frailty could potentially be developed earlier.

### Strengths and limitations

The strengths of the study included a large patient dataset along with long-term outcomes. Unlike previous evidence for HFRS, to the best of our knowledge, this was the first study to use ICD-10 codes only from the pre-existing conditions and not illnesses that might have developed (and coded) during the index hospitalisation. A few limitations need to be acknowledged. Firstly, the CFS measures patients’ frailty in the 2 months before ICU admission, however, the derived HFRS used in our study for only pre-existing conditions may pre-date index hospitalisation, and hence comparable with CFS. Secondly, there could be a possibility of inappropriate categorisation, which may be a consequence of how the data is collected. For example, historically, elective surgical patients are more likely to have comorbidities listed because they often have had a preoperative workup, while the past medical histories for emergency patients were often incomplete [34]. Although the HFRS is calculated at the time of discharge, sensitivity analysis on elective and non-elective admissions suggested that under-coding of comorbidities happened with non-elective admissions. This could have affected the HFRS scores and therefore the frailty status of patients [35]. Thirdly, with the advent of population and disease registers, more studies rely on such registries for gathering vital information; hence inadvertently making them prone to information bias due to either inaccurate measurement or documentation [36]. Fourthly, we were unable to determine inter-rater reliability for CFS in our study. However, previous studies investigating the inter-rater reliability of the CFS in critically ill patients showed that the inter-rater reliability was strong when intensive care clinicians assessed the CFS [37]. However, we were unable to determine inter-rater reliability for CFS in our study. Fifthly, not all data from the ANZICS-APD with the CFS were linked to VAED and VDI datasets. Sixthly, there was a significant variation in the CFS documentation (ranging between 7 and 100%) in the 16 hospitals that reported on the CFS with documentation of the CFS increasing over time (17% at commencement, 85% at completion; Additional file 1: Table S8). However, given there was little clinical difference between included and missing patient data (*n* = 13,457), our cohort was broadly representative of the larger population. Finally, the lower ICU mortality rates and short ICU length of stays are real-world outcomes for ANZ ICUs and may not be applicable to other countries.

## Conclusion

This retrospective multicentre cohort study in critically ill patients found that although both frailty measures independently predicted 1-year survival with moderate discrimination, the CFS was a better predictor of 1-year survival than the HFRS. The findings also suggest that the CFS had greater validity, particularly in mechanically ventilated patients and those who were admitted non-electively compared to HFRS. However, neither frailty measure was able to clinically improve upon the predictability provided by baseline patient illness severity as assessed by ANZROD.

## Supplementary Information

**Additional file 1.** **Supplementary Table 1:** ICD-10 codes used to estimate the HFRS, and the points awarded for each diagnosis. **Supplementary Table 2:** A complete list of ICD-10 variables extracted from the VAED database. **Supplementary Table 3:** A complete list of ICD-10 variables extracted from the VDI database. **Supplementary Table 4:** Comparison between included (n=7,001) vs excluded (n = 13,457). **Supplementary Table 5:** Spearman correlation and Kappa agreement between the two frailty measures. **Supplementary Table 6:** Subgroup analysis: Unadjusted and adjusted Cox proportional hazards regression for CFS and the HFRS as continuous variables, adjusting for ANZROD, and sex CFS and HFRS or (a) Patients ≥ 65 years of age, (b) Patients ≥75 years of age, (c) Patients needing mechanical ventilation, (d) elective patients, (e) non-elective patients and (f) patients who survived to hospital discharge. **Supplementary Table 7:** Multivariable Logistic Regression analysis for short-term and long-term mortalities. Unadjusted and adjusted for sex and ANZROD. The odds ratio (OR) in bold represent statistically significant results. Graphically presented in Fig. 2. **Supplementary Table 8:** Reduction over time in the proportion of patients without a documented CFS in the ANZICS database. **Supplementary Figure 1:** Prevalence of frailty measure by CFS and HFRS across different age categories. **Supplementary Figure 2:** Kaplan Meier curves between frail and non-frail patients (treated as a dichotomous variable) for CFS and HFRS. **Supplementary Figure 3:** Area under the receiver operator curve for short-term mortality. The CFS was significantly better than the HFRS. However, CFS and HFRS must both be measuring different aspects, because the combination of the two was better than either measure on its own. The bottom 2 graphs showed that while both variables may be predictive of mortality, neither provided any worthwhile improvement on top of ANZROD. **Supplementary Figure 4:** Area under the receiver operator curve for 1-year mortality for patients ≥ 75 years of age and those needing mechanical ventilation.

## Data Availability

The datasets generated and/or analysed during the current study are not publicly available as these are linked from three registries (ANZICS, VAED and VDI), but are available from the corresponding author on reasonable request.

## Abbreviations

ANZICS: Australia and New Zealand Intensive Care Society
ANZm: ANZROD + male sex
ANZROD: Australia and New Zealand risk of death
APACHE: Acute Physiology and Chronic Health Evaluation
APD: Adult Patient Database
AR-DRG: Australian-refined Diagnosis-related groups
AUROC: Area under the receiver operating characteristic
COPD: Chronic obstructive pulmonary disease
CFS: Clinical Frailty Scale
DHHS: Department of Health and Human Services
HFRS: Hospital frailty risk score
HR: Hazard ratio
HREC: Hospital Research ethics committee
ICD-10: International Statistical Classification of Diseases and Related Health Problems, 10th Revision
ICU: Intensive care unit
IQR: Interquartile range
LOS: Length of stay
n: Number
NYHA: New York Heart Association
OR: Odds ratio
RACF: Residential aged care facility
RoD: Risk of death
RRT: Renal replacement therapy
SAPS 3: Simplified Acute Physiology admission score
SD: Standard deviation
SOFA: Sequential organ failure assessment
T_COV_: Time-dependent covariate
TCP: Transitional care program
VAED: Victorian admitted episode database
VDI: Victorian death index
