# Supplementary material for: Comparison of the predictive ability of clinical frailty scale and hospital frailty risk score to determine long-term survival in critically ill patients: a multicentre retrospective cohort study
Source: Crit Care. 2022 May 3;26:121. doi: 10.1186/s13054-022-03987-1 (PMC9063154; doi:10.1186/s13054-022-03987-1)
Supplement: Supplementary file 1 — Additional file 1. Supplementary Table 1: ICD-10 codes used to estimate the HFRS, and the points awarded for each diagnosis. Supplementary Table 2: A complete list of ICD-10 variables extracted from the VAED database. Supplementary Table 3: A complete list of ICD-10 variables extracted from the VDI database. Supplementary Table 4: Comparison between included (n=7,001) vs excluded (n = 13,457). Supplementary Table 5: Spearman correlation and Kappa agreement between the two frailty measures. Supplementary Table 6: Subgroup analysis: Unadjusted and adjusted Cox proportional hazards regression for CFS and the HFRS as continuous variables, adjusting for ANZROD, and sex CFS and HFRS or (a) Patients ≥ 65 years of age, (b) Patients ≥75 years of age, (c) Patients needing mechanical ventilation, (d) elective patients, (e) non-elective patients and (f) patients who survived to hospital discharge. Supplementary Table 7: Multivariable Logistic Regression analysis for short-term and long-term mortalities. Unadjusted and adjusted for sex and ANZROD. The odds ratio (OR) in bold represent statistically significant results. Graphically presented in Fig. 2. Supplementary Table 8: Reduction over time in the proportion of patients without a documented CFS in the ANZICS database. Supplementary Figure 1: Prevalence of frailty measure by CFS and HFRS across different age categories. Supplementary Figure 2: Kaplan Meier curves between frail and non-frail patients (treated as a dichotomous variable) for CFS and HFRS. Supplementary Figure 3: Area under the receiver operator curve for short-term mortality. The CFS was significantly better than the HFRS. However, CFS and HFRS must both be measuring different aspects, because the combination of the two was better than either measure on its own. The bottom 2 graphs showed that while both variables may be predictive of mortality, neither provided any worthwhile improvement on top of ANZROD. Supplementary Figure 4: Area under the receiver operator [file 13054_2022_3987_MOESM1_ESM.docx]

**Legends for Supplementary** **Tables and Figures**

**Supplementary Table 1:** ICD-10 codes used to estimate the HFRS, and the points awarded for each diagnosis.

**Supplementary Table 2:** A complete list of ICD-10 variables extracted from the VAED database.

**Supplementary Table 3:** A complete list of ICD-10 variables extracted from the VDI database.

**Supplementary Table 4:** Comparison between included (n=7,001) vs excluded (n=13,457).

**Supplementary Table 5:** Spearman correlation and Kappa agreement between the two frailty measures.

**Supplementary Table 6:** Subgroup analysis: Unadjusted and adjusted Cox proportional hazards regression for CFS and the HFRS as continuous variables, adjusting for ANZROD, and sex CFS and HFRS or (a) Patients ≥65 years of age, (b) Patients ≥75 years of age, (c) Patients needing mechanical ventilation, (d) elective patients, (e) non-elective patients and (f) patients who survived to hospital discharge.

**Supplementary Table 7:** Multivariable Logistic Regression analysis for short-term and long-term mortalities. Unadjusted and adjusted for sex and ANZROD. The odds ratio (OR) in bold represent statistically significant results. Graphically presented in Figure 2.

**Supplementary Table 8:** Reduction over time in the proportion of patients without a documented CFS in the ANZICS database.

**Supplementary Figure 1:** Prevalence of frailty measure by CFS and HFRS across different age categories.

**Supplementary Figure 2:** Kaplan Meier curves between frail and non-frail patients (treated as a dichotomous variable) for CFS and HFRS.

**Supplementary Figure 3:** Area under the receiver operator curve for short-term mortality. The CFS was significantly better than the HFRS. However, CFS and HFRS must both be measuring different aspects, because the combination of the two was better than either measure on its own. The bottom 2 graphs showed that while both variables may be predictive of mortality, neither provided any worthwhile improvement on top of ANZROD.

**Supplementary Figure 4:** Area under the receiver operator curve for 1-year mortality for patients ≥75 years of age and those needing mechanical ventilation.

**Supplementary Table 1:** ICD-10 codes used to estimate the HFRS, and the points awarded for each diagnosis.

| **ICD-10 Code** | **Diagnosis** | **Points Awarded** | **Frequency** | **Percentage (%)** |
| --- | --- | --- | --- | --- |
| N17 | Acute renal failure | 1·8 | 1023 | 14.6 |
| E87 | Other disorders of fluid, electrolyte, and acid-base balance | 2·3 | 940 | 13.4 |
| A41 | Other septicaemia | 1·6 | 660 | 9.4 |
| N18 | Chronic renal failure | 1·4 | 608 | 8.7 |
| I95 | Hypotension | 1·6 | 583 | 8.3 |
| D64 | Other anaemias | 0·4 | 458 | 6.5 |
| J96 | Respiratory failure, not elsewhere classified | 1·5 | 422 | 6.0 |
| S22 | Fracture of rib(s), sternum, and thoracic spine | 1·8 | 411 | 5.9 |
| F10 | Mental and behavioural disorders due to use of alcohol | 0·7 | 344 | 4.9 |
| J18 | Pneumonia, organism unspecified | 1·1 | 331 | 4.7 |
| B96 | Other bacterial agents as the cause of diseases classified to other chapters (secondary code) | 2·9 | 311 | 4.4 |
| E83 | Disorders of mineral metabolism | 0·4 | 290 | 4.1 |
| S06 | Intracranial injury | 2·4 | 289 | 4.1 |
| B95 | Streptococcus and staphylococcus as the cause of diseases classified to other chapters | 1·7 | 230 | 3.3 |
| R13 | Dysphagia | 0·8 | 228 | 3.3 |
| N39 | Other disorders of the urinary system (includes urinary tract infection and urinary incontinence) | 3·2 | 226 | 3.2 |
| F05 | Delirium, not induced by alcohol and other psychoactive substances | 3·2 | 200 | 2.9 |
| S01 | Open wound of head | 1·1 | 191 | 2.7 |
| U80 | Agent resistant to penicillin and related antibiotics | 0·8 | 191 | 2.7 |
| E86 | Volume depletion | 2·3 | 182 | 2.6 |
| S32 | Fracture of lumbar spine and pelvis | 1·4 | 178 | 2.5 |
| J69 | Pneumonitis due to solids and liquids | 1·0 | 177 | 2.5 |
| R41 | Other symptoms and signs involving cognitive functions and awareness | 2·7 | 159 | 2.3 |
| L89 | Decubitus ulcer | 1·7 | 149 | 2.1 |
| R94 | Abnormal results of function studies | 1·4 | 146 | 2.1 |
| R00 | Abnormalities of heartbeat | 0·7 | 145 | 2.1 |
| Z91 | Personal history of risk factors, not elsewhere classified | 0·5 | 145 | 2.1 |
| K59 | Other functional intestinal disorders | 1·8 | 138 | 2.0 |
| S72 | Fracture of femur | 1·4 | 128 | 1.8 |
| F32 | Depressive episode | 0·5 | 127 | 1.8 |
| S42 | Fracture of shoulder and upper arm | 2·3 | 125 | 1.8 |
| W19 | Unspecified fall | 3·2 | 116 | 1.7 |
| A09 | Diarrhoea and gastroenteritis of presumed infectious origin | 1·1 | 116 | 1.7 |
| K92 | Other diseases of the digestive system | 0·8 | 111 | 1.6 |
| G81 | Hemiplegia | 4·4 | 109 | 1.6 |
| R40 | Somnolence, stupor and coma | 2·5 | 107 | 1.5 |
| I63 | Cerebral Infarction | 0·8 | 105 | 1.5 |
| L03 | Cellulitis | 2·0 | 103 | 1.5 |
| S80 | Superficial injury of the lower leg | 2·0 | 102 | 1.5 |
| R45 | Symptoms and signs involving emotional state | 1·2 | 102 | 1.5 |
| R47 | Speech disturbances, not elsewhere classified | 1·0 | 101 | 1.4 |
| S00 | Superficial injury of head | 3·2 | 99 | 1.4 |
| R56 | Convulsions, not elsewhere classified | 2·6 | 98 | 1.4 |
| W18 | Other fall on the same level | 2·1 | 92 | 1.3 |
| Y84 | Other medical procedures as the cause of abnormal reaction of the patient | 0·7 | 92 | 1.3 |
| R79 | Other abnormal findings of blood chemistry | 0·6 | 76 | 1.1 |
| R50 | Fever of unknown origin | 0·1 | 76 | 1.1 |
| X59 | Exposure to unspecified factor | 1·5 | 74 | 1.1 |
| R63 | Symptoms and signs concerning food and fluid intake | 0·9 | 74 | 1.1 |
| W01 | Fall on the same level from slipping, tripping and stumbling | 0·9 | 73 | 1.0 |
| S51 | Open wound of forearm | 0·5 | 72 | 1.0 |
| J22 | Unspecified acute lower respiratory infection | 0·7 | 63 | 0.9 |
| R33 | Retention of urine | 1·3 | 60 | 0.9 |
| R11 | Nausea and vomiting | 0·3 | 60 | 0.9 |
| R55 | Syncope and collapse | 1·8 | 57 | 0.8 |
| Z60 | Problems related to social environment | 1·8 | 54 | 0.8 |
| M25 | Other joint disorders, not elsewhere classified | 2·3 | 52 | 0.7 |
| Y95 | Nosocomial condition | 1·2 | 51 | 0.7 |
| K26 | Duodenal ulcer | 1·6 | 47 | 0.7 |
| G40 | Epilepsy | 1·5 | 44 | 0.6 |
| R29.6 | Tendency to fall | 3·6 | 42 | 0.6 |
| R31 | Unspecified haematuria | 3·0 | 40 | 0.6 |
| M79 | Other soft tissue disorders, not elsewhere classified | 1·1 | 39 | 0.6 |
| Z22 | Carrier of infectious disease | 1·7 | 36 | 0.5 |
| L97 | Ulcer of the lower limb, not elsewhere classified | 1·6 | 35 | 0.5 |
| R02 | Gangrene, not elsewhere classified | 1·0 | 34 | 0.5 |
| N28 | Other disorders of kidney and ureter, not elsewhere classified | 1·3 | 32 | 0.5 |
| M48 | Spinal stenosis (secondary code only) | 0·5 | 30 | 0.4 |
| R26 | Abnormalities of gait and mobility | 2·6 | 29 | 0.4 |
| I67 | Other cerebrovascular diseases | 2·6 | 28 | 0.4 |
| E16 | Other disorders of pancreatic internal secretion | 1·4 | 28 | 0.4 |
| A04 | Other bacterial intestinal infections | 1·1 | 28 | 0.4 |
| W10 | Fall on and from stairs and steps | 0·9 | 28 | 0.4 |
| E05 | Thyrotoxicosis [hyperthyroidism] | 0·9 | 27 | 0.4 |
| Z75 | Problems related to medical facilities and other health care | 2·0 | 23 | 0.3 |
| R32 | Unspecified urinary incontinence | 1·2 | 23 | 0.3 |
| K52 | Other noninfective gastroenteritis and colitis | 0·3 | 23 | 0.3 |
| I69 | Sequelae of cerebrovascular disease (secondary codes) | 3·7 | 22 | 0.3 |
| T83 | Complications of genitourinary prosthetic devices, implants and grafts | 2·4 | 21 | 0.3 |
| S09 | Other and unspecified injuries of the head | 1·2 | 21 | 0.3 |
| W06 | Fall involving bed | 1·1 | 18 | 0.3 |
| M19 | Other arthrosis | 1·5 | 15 | 0.2 |
| M81 | Osteoporosis without pathological fracture | 1·4 | 15 | 0.2 |
| N20 | Calculus of kidney and ureter | 0·7 | 15 | 0.2 |
| G45 | Transient cerebral ischaemic attacks and related syndromes | 1·2 | 13 | 0.2 |
| R44 | Other symptoms and signs involving general sensations and perceptions | 1·6 | 12 | 0.2 |
| G20 | Parkinson's disease | 1·8 | 11 | 0.2 |
| E55 | Vitamin D deficiency | 1·0 | 11 | 0.2 |
| H91 | Other hearing loss | 0·9 | 11 | 0.2 |
| F03 | Unspecified dementia | 2·1 | 10 | 0.1 |
| N19 | Unspecified renal failure | 1·6 | 10 | 0.1 |
| Z93 | Artificial opening status | 1·0 | 10 | 0.1 |
| Z99 | Dependence on enabling machines and devices | 0·8 | 10 | 0.1 |
| M41 | Scoliosis | 0·9 | 9 | 0.1 |
| L08 | Other local infections of the skin and subcutaneous tissue | 0·4 | 8 | 0.1 |
| E53 | Deficiency of other B group vitamins | 1·9 | 7 | 0.1 |
| F00 | Dementia in Alzheimer's disease | 7·1 | 5 | 0.1 |
| G30 | Alzheimer's disease | 4·0 | 5 | 0.1 |
| Z74 | Problems related to care-provider dependency | 1·1 | 4 | 0.1 |
| Z50 | Care involving the use of rehabilitation procedures | 2·1 | 3 | 0.0 |
| F01 | Vascular dementia | 2·0 | 3 | 0.0 |
| M80 | Osteoporosis with pathological fracture | 0·8 | 3 | 0.0 |
| H54 | Blindness and low vision | 1·9 | 2 | 0.0 |
| Z87 | Personal history of other diseases and conditions | 1·5 | 1 | 0.0 |
| Z73 | Problems related to life-management difficulty | 0·6 | 1 | 0.0 |
| M15 | Polyarthrosis | 0·4 | 1 | 0.0 |
| R54 | Senility | 2·2 | 0 | 0.0 |
| R69 | Unknown and unspecified causes of morbidity | 1·3 | 0 | 0.0 |
| G31 | Other degenerative diseases of the nervous system, not elsewhere classified | 1·2 | 0 | 0.0 |

**Supplementary Table 2:** A complete list of ICD-10 variables extracted from the VAED database.

| **Data Field Description** | **Definition** |
| --- | --- |
| Month and Year of Admission | Month and Year on which a patient commences an episode of care. |
| Care type | The nature of clinical service (type of care) provided to an admitted patient during an episode of care. There must be one and only one care type code per episode. A change in care type results in a statistical separation and a new episode with a statis |
|  | 1 NHT/Non-Acute |
|  | P Designated Paediatric Rehabilitation Program/Unit |
|  | 2 Designated Rehabilitation Program/Unit: Level 1 |
|  | 6 Designated Rehabilitation Program/Unit: Level 2 |
|  | 8 Palliative Care Program |
|  | 5 Mental Health |
|  | 9 Geriatric Evaluation and Management Program |
|  | R1 Restorative Care: On-site |
|  | R2 Restorative Care: Off-site |
|  | 0 Alcohol and Drug Program |
|  | 4 Other care (Acute) including Qualified newborn |
|  | U Unqualified newborn |
|  | N Qualified Newborn |
|  | U Unqualified Newborn |
|  | X Not Applicable |
| Admission type | The type of admission relating to this episode of care: |
|  | K Posthumous Organ Procurement |
|  | S Statistical admission (change in Care Type within this hospital) |
|  | Y Birth episode |
|  | M Maternity |
|  | C Emergency admission through Emergency Department at this hospital (VEMD reporting hospitals only) |
|  | L Admission – from the Waiting List (ESIS reporting hospitals only) |
|  | O Other emergency admission |
|  | X Other admission |
| Admission type indicator | Admission type indicator derived from Admission Type |
|  | E Emergency |
|  | L Elective |
|  | M Maternity |
|  | N Newborn (<= 9 days old) |
|  | S Statistical |
| Criterion for Admission | This field indicates the criterion for admission for the episode of care. |
|  | K Posthumous Organ Procurement |
|  | N Qualified newborn |
|  | U Unqualified newborn |
|  | R Restorative Care: Off-site |
|  | O Patient expected to require hospitalisation for a minimum of one night |
|  | B Day-only Automatically Admitted Procedures |
|  | E Day-only Extended Medical Treatment |
|  | C Day-only Not Automatically Qualified Procedures |
|  | S Secondary family member |
| Admission/readmission to rehabilitation | For Care Types P, 2 and 6, this field indicates whether this is the first or subsequent rehabilitation episode for a particular injury/condition. |
|  | 0 First rehabilitation admission |
|  | 1 Readmission for rehabilitation |
| RUG ADL on admission | RUG ADL (Resource Utilisation Groups Activities of Daily Living) score as assessed on admission. Cumulative score out of 18. |
| Source of referral to palliative care | Source of referral to the DH Palliative Care Program (Care Type 8). |
|  | 01 Community sector – GP |
|  | 02 Community sector – Specialist |
|  | 03 Community sector - Self, Carer, Other (family member, neighbour) |
|  | 04 Community sector- Community-based agency |
|  | 05 Hospital - Public - Admitted patient |
|  | 06 Hospital - Private - Admitted patient |
|  | 07 Hospital - Outpatient - Non-admitted |
|  | 08 Residential care - Nursing home/hostel |
|  | 09 Other |
| 5 year age groups | Five-year age groups |
|  | 00‑04 05‑09 10‑14 15‑19 20‑24 |
|  | 25‑29 30‑34 35‑39 40‑44 45‑49 |
|  | 50‑54 55‑59 60‑64 65‑69 70‑74 |
|  | 75‑79 80‑84 85+ |
| Sex of patient | The sex of the patient: |
|  | 1 Male |
|  | 2 Female |
|  | 3 Indeterminate (only for infants < 90 days old) |
|  | 4 Intersex |
| Statistical local area (5 digits) | The patient’s Statistical Local Area of residence. Based on Australian Standard Geographical Classification (ASGC) 2011 boundaries and derived from the locality and postcode. |
| Local government area | The patient’s Local Government Area of residence. Based on Australian Standard Geographical Classification (ASGC) 2004 boundaries for Victoria, and 1999 boundaries for the rest of Australia. |
| Region of residence | The code for the Department of Health/Human Services Region in which the patient resides; is derived from the field ‘SLA’. |
|  | 1 Barwon Southwestern |
|  | 2 Grampians |
|  | 3 Loddon Mallee |
|  | 4 Hume |
|  | 5 Gippsland |
|  | 8 Eastern |
|  | 9 Southern |
|  | A North-Western |
|  | I Interstate |
|  | M Missing |
| State of residence | State of patient residence derived from SLA: |
|  | 0 Unknown/Itinerate/Overseas |
|  | 1 New South Wales |
|  | 2 Victoria |
|  | 3 Queensland |
|  | 4 South Australia |
|  | 5 Western Australia |
|  | 6 Tasmania |
|  | 7 Northern Territory |
|  | 8 Australian Capital Territory |
|  | 9 Other Territories |
| Carer availability | A record of whether a person, such as a family member, friend or neighbour has been identified as providing regular ongoing care or assistance, which is not linked to a formal service. |
|  | 1 Carer not needed/ not applicable |
|  | 2 Lives alone, has a carer |
|  | 3 Lives alone, has no carer |
|  | 4 Lives with another, has no carer |
|  | 5 Lives with another, has a resident carer |
|  | 6 Lives with another, has a non-resident carer |
|  | 7 Lives in a mutually dependent situation |
|  | 8 Missing or not recorded |
| Hospital Region | Metropolitan/Rural flag of hospitals. |
| Month and Year of separation | Month and Year of separation, eg. Jul, Aug |
| Length of stay | The length of stay is calculated during the PRS/2 processing, summing the total patient days in each of the status segments minus leave with and without permission days. |
| Hospital in the home length of stay | Hospital in the Home Length of Stay. |
| Hospital in the Home separation | Flag to indicate that the episode includes a “Hospital In The Home” component. |
| Patient type | Patient type derived from Separation Account: |
|  | H Public |
|  | P Private |
|  | S Compensable |
|  | V DVA |
|  | X Ineligible |
| Duration of unit stay | Identifies the duration of stay within a specific campus unit. |
|  | E Entire admission was at the specified campus unit |
|  | P Part of the admission was at the specified campus unit. |
| Barthel index on separation | The Barthel Index on separation is assessed on the day on which the decision is taken to cease rehabilitation (for Care Types F, E, 2, 6, 7, 9 or K). |
| RUG ADL on separation | RUGADL (Resource Utilisation Groups Activities of Daily Living) score as assessed on separation (for Care Type 8). Cumulative score out of 18. |
| Victorian Adjusted AR-DRGv6 | Victorian Adjusted Australian Revised Diagnosis Related Group v6 is the same as AR-DRG v6 except where adjustments are made utilising the VIC-DRG6 field, for case-mix payments. |
| Victorian adjusted AR-MDCv6 | The Australian Revised Major Diagnostic Category (AR-MDC) Version 6.0 is derived through the same grouping process as the AR-DRG v6. |
| Clinical speciality | Clinical speciality mapped from VIC. DRG v6.0x (665 DRGs mapped into 27 Clinical Specialties): |
|  | 01 Neurosurgery |
|  | 03 Vascular |
|  | 04 Orthopaedics |
|  | 05 Neurology |
|  | 06 Ophthalmology |
|  | 07 ENT |
|  | 08 Cardiothoracic |
|  | 09 Cardiology |
|  | 10 Rehabilitation |
|  | 11 Dental |
|  | 12 Rheumatology |
|  | 13 Plastics |
|  | 14 General Medicine |
|  | 15 Psychiatry |
|  | 16 General Surgery |
|  | 17 Nephrology |
|  | 18 Renal Dialysis |
|  | 19 Urology |
|  | 20 Gynaecology |
|  | 21 Obstetrics & Ante-natal |
|  | 22 Neonatology |
|  | 23 Haematology |
|  | 24 Respiratory |
|  | 25 Oncology/Radiology |
|  | 26 Endocrinology |
|  | 27 Gastroenterology |
|  | 28 Other/Ungroupable |
| DRG Type | DRG type: |
|  | M Medical |
|  | S Surgical |
|  | O Other |
| DRG Coding status | Coding status of separation records: |
|  | C Coded |
|  | P Problem DRG (AR-DRG 6.0: 801A, 801B, 801C) |
|  | <Blank> Not Coded |
| First external-cause activity | The first diagnosis code is in the range U50 – U73. |
| First external-cause place of occurrence | The first diagnosis code commencing with Y92. |
| Principal external-cause | If the first diagnosis is an injury or poisoning, i.e. in the range S00 to T98, then the principal external cause is the first code in the range of V01 to Y91 or Y95 to Y98. |
| Principal external-cause activity | If the first diagnosis is an injury or poisoning i.e. in the range S00 to T98, and principal external cause in range V01-Y34, then the activity is the first diagnosis code in the range U50 – U73. |
| Principal external-cause place of occurrence | If the first diagnosis is an injury or poisoning, i.e. in the range S00 to T98, and principal external cause in range V01 – Y89, then “place of occurrence” is the first diagnosis code commencing with Y92. |
| Duration of stay (hours) in the intensive care unit | The total duration of stay (hours) in an approved Intensive Care Unit (ICU) or Neonatal Intensive Care Unit (NICU), during this episode of care. |
|  | Duration is reported in hours, rounded up to the nearest hour. |
| Duration of Mechanical Ventilation in ICU | The total duration of Mechanical Ventilation (MV) in hours provided in an approved Intensive Care Unit (ICU) or Neonatal Intensive Care (NICU) during this episode of care. |
| Duration of Non-Invasive Ventilation | The total number of hours of non-invasive ventilatory assistance given via any route other than intubation or tracheostomy, provided to patients in an approved Neonatal Intensive Care Unit (NICU) or Special Care Nursery (SCN) or Intensive Care Unit (ICU). |
| Clinical Sub-program | The diagnosis is based on the body system manifesting the reason for rehabilitation. Reported for Care Types 2, 6, 7, K and P. Clinical Sub-Program is assigned by the treating clinician. |
| Impairment | A code assigned, based on the body system manifesting the reason for rehabilitation. Only reported for Sub-acute records. Reported for Care Type 2,6, P, R1 and R2. Introduction of Version 1 Australian Impairment code-set for Sub-Acute episodes as an optional field. |
|  |  |
| Mental Health legal status | A funding-source indicator for involuntary patients: |
|  | 1 Involuntary for all or part of this episode |
|  | 2 Not involuntary at any time during this episode |
|  | 9 Not Applicable |
| Admission Source | Describes where the patient was residing or living before the commencement of an episode of care. |
|  | A Transfer from mental health residential facility |
|  | B Transfer from Transition Care bed-based program |
|  | H Admission from private residence/ accommodation |
|  | N Transfer from aged care residential facility |
|  | S Statistical Admission (change in Care Type within this hospital) |
|  | T* Transfer from acute/ extended care/ rehabilitation/ geriatric centre |
|  | ***** Requires an admission transfer code |
|  | Y Birth Episode |
| Encrypted Campus code | Indicates the hospital campus where the episode of care was provided. The patient activity must be reported under the campus code at which it occurred. |
| Interpreter Required | The patient’s need for an interpreter, as perceived by the patient or person consenting for the patient. |
|  | 1 Yes |
|  | 2 No |
|  | 9 Not Stated/Inadequately described |
| Marital status | The current marital or living status of the patient at the time of admission: |
|  | 1 Never married |
|  | 2 Widowed |
|  | 3 Divorced |
|  | 4 Separated |
|  | 5 Married |
|  | 6 De Facto |
|  | 9 Not stated/inadequately described |
| Preferred Language | The language (including sign language) is most preferred by the patient for communication. This may be a language other than English even where the person can speak fluent English. |
| Indigenous status | The indigenous status of the patient as determined by patient self-identification. |
| Encrypted Transfer Source (FROM) | Identification of the hospital campus the person has been transferred from, following separation from that hospital. |
|  |  |
| Account class on separation | The patient account classification on separation. |
| Encrypted Transfer destination (TO) | Identification of the hospital campus to which the patient is transferred after separation from this hospital. |
|  | Y Yes |
|  | N No |
|  | U Uncoded (but eligible for WIES funding when coded) |
| Separation Mode | Type of separation: |
|  | A Separation and transfer to mental health residential facility |
|  | B Separation and transfer to Transition Care bed-based program |
|  | D Death |
|  | H Separation to private residence/accommodation |
|  | S Statistical Separation |
|  | N Separation and transfer to aged care residential facility |
|  | T* Separation and transfer to other acute hospital/extended care/rehabilitation/geriatric centre |
|  | R Separation and transfer to Restorative Care bed- based program |
|  | ***** Requires separation transfer code |
|  | Z Left against medical advice |
| Separation referral | Clinical care and support services arranged by the hospital to meet the person’s recuperative needs when discharged to private accommodation or home. Up to four referrals can be transmitted in the one field. |
|  | A Referral to Aged Care Assessment Service (ACAS), arranged before discharge |
|  | B Community palliative care support arranged before discharge |
|  | C Mental health community services arranged before discharge |
|  | D Psychiatric disability support services arranged before discharge |
|  | F Domiciliary postnatal care arranged before discharge |
|  | G Referral to general practitioner arranged before discharge |
|  | K Referral to Aboriginal and Torres Strait Islander (ATSI) service, arranged before discharge |
|  | L Alcohol and drug treatment service arranged before discharge |
|  | M Referral to a community rehab centre arranged before discharge |
|  | P Post-Acute Care Program services arranged before discharge |
|  | R Other clinical care &/or support services arranged before discharge |
|  | S Referral to private psychiatrist arranged before discharge |
|  | T Referral to Transition Care home-based program, arranged before discharge |
|  | U Home nursing support arranged before discharge |
|  | X No referral or support services arranged before discharge. |

**Supplementary Table 3:** A complete list of ICD-10 variables extracted from the VDI database.

| **Data Field Description** | **Justification** |
| --- | --- |
| Cause of Death |  |
| Deceased Date of Death (Month/year) | Estimation of long-term survival after ICU admission |
| Deceased Age at Death (Month/year) | Estimation of long-term survival after ICU admission |

**Supplementary Table 4:** Comparison between included (n=7,001) vs excluded (n=13,457).

|  | **Overall** | **Excluded Patients** | **Included Patients** | **p-value** |  |
| --- | --- | --- | --- | --- | --- |
| **Number of patients,** % (n) | 20458 | 13457 | 7001 | - |  |
| **Age (years),** mean (SD) | 61.8 (17.6) | 62.5 (17.5) | 60.5 (17.6) | <0.0001 |  |
| **Male**, % (n) | 57.0 (11689) | 55.9 (7523) | 59.5 (4166) | <0.0001 |  |
| **Clinical Frailty Scale score,** mean (SD) | 3.4 (1.5) | 3.5 (1.9) (2439) | 3.3 (1.5) | <0.0001 |  |
| **ICU Admission Type**, % (n) | | | | | |
| -        Elective Surgery | 23.8 (4877) | 23.3 (3137) | 24.9 (1740) | 0.014 |  |
| **Comorbidities**, % (n) | | | | | |
| -        Chronic respiratory disorder | 8.5 (1741) | 8.9 (1202) | 7.7 (539) | 0.003 |  |
| -        Cardiovascular disorder | 8.6 (1757) | 9.6 (1287) | 6.7 (470) | <0.0001 |  |
| -        Chronic renal failure | 4.3 (874) | 4.6 (614) | 3.7 (260) | 0.004 |  |
| -        Hepatic failure | 2.6 (536) | 2.6 (355) | 2.6 (181) | 0.82 |  |
| -        Cirrhosis | 2.6 (539) | 2.7 (358) | 2.6 (181) | 0.75 |  |
| -        Immune disorders | 1.7 (351) | 1.5 (200) | 2.2 (151) | <0.0001 |  |
| -        Immunodeficiency | 5 (1020) | 4.6 (625) | 5.6 (395) | 0.002 |  |
| -        Metastatic cancer | 3 (623) | 3.3 (440) | 2.6 (183) | 0.001 |  |
| -        Lymphoma | 1.1 (218) | 1.2 (166) | 0.7 (52) | 0.001 |  |
| -        Leukaemia | 1.4 (279) | 1.2 (168) | 1.6 (111) | 0.049 |  |
| Charlson Comorbidity Index | 0 (0-2) | 0 (0-2) | 0 (0-2) | 0.85 |  |
| Pre-ICU (Hours), median (IQR) | 8 (4-22.2) | 8.3 (4.1-22.8) | 7.5 (3.8-20.1) | <0.0001 |  |
| **Scoring and Risk of Death scores** | | | | | |
| -        APACHE-2 score, mean (SD) | 15.6 (7.8) | 15.7 (8.0) | 15.4 (7.5) | 0.034 |  |
| -        APACHE-3 score, mean (SD) | 52.4 (26.2) | 52.6 (26.9) | 51.9 (24.7) | 0.06 |  |
| -        ANZROD Score, (%) median (IQR) | 2.7 (0.8-10) | 2.9 (0.8-10) | 2.3 (0.7-9.1) | <0.0001 |  |
| **Organ failure and supports**, % (n) | | | | | |
| -        Invasive ventilation | 38.4 (7858) | 34.1 (4592) | 46.7 (3266) | <0.0001 |  |
| -        Renal replacement therapy | 3.3 (671) | 2.3 (314) | 5.1 (357) | <0.0001 |  |
| **Mortality**, % (n) | | | | | |
| -        ICU Mortality | 6.5 (1328) | 6.4 (856) | 6.7 (472) | 0.30 |  |
| -        Hospital Mortality | 9.4 (1924) | 9.5 (1282) | 9.2 (642) | 0.39 |  |
| -        12-month Mortality | 14.6 (2995) | 14.8 (1989) | 14.4 (1006) | 0.43 |  |
| **Length of Stay**, median (IQR) | | | | | |
| -        ICU length of stay (hours) | 46 (24-89) | 44 (23-84) | 47 (24-93) | <0.0001 |  |
| -        Hospital length of stay (days) | 7 (4-14) | 7 (4-13) | 8 (4-15) | <0.0001 |  |
| ICU >10 days | 5.2 (1057) | 4.6 (617) | 6.3 (440) | <0.0001 |  |
| Readmission | 4.0 (823) | 4.1 (547) | 3.9 (276) | 0.67 |  |
| **Discharge Destination**, % (n) | | | | | |
| -        Usual residence | 62.3 (12745) | 56.9 (7657) | 64.2 (4496) | <0.0001 |  |
| SD = standard deviation; n = number; IQR = interquartile range; ICU = intensive care unit; ICU – intensive care unit; SD – standard deviation; APACHE - Acute Physiology and Chronic Health Evaluation | | | | | |

**Supplementary Table 5:** Spearman correlation and Kappa agreement between the two frailty measures.

| **HFRS** | **CFS score** | | **Spearman's Correlation^(1)^** | **Agreement^(2)^** |
| --- | --- | --- | --- | --- |
|  | **non-frail**  **(CFS <5)** | **Frail**  **(CFS≥5)** | **Correlation coefficient**  **(95%-CI; p-value)** | **Kappa**  **(95%-CI; p-value)** |
| **Patients ≥75 years of age (n=1683)** | **1164** | **519** |  | |
| HFRS (non-frail; n=1151 [68.4%]) | 859 | 292 | 0.22  (0.18-0.27; p<0.001) | 0.17  (0.12-0.19; p<0.001) |
| HFRS (frail; n=532 [31.6%]) | 305 | 227 |  |  |
| **Mechanical ventilation (n=3266)** | **2722** | **544** |  | |
| HFRS (non-frail; n=2414 [73.9%]) | 2039 | 375 | -0.02  (-0.05-0.02; p=0.40) | 0.05  (0.01-0.08; p=0.004) |
| HFRS (frail; n=852 [26.1%]) | 683 | 169 |  |  |
| **Elective Patients (n=1740)** | 1533 | 207 |  | |
| HFRS (non-frail; n=1670 [96.0%]) | 1485 | 185 | 0.17  (0.12-0.22; p<0.001) | 0.11  (0.06-0.14; p<0.001) |
| HFRS (frail; n=70 [4.0%]) | 48 | 22 |  |  |
| **Non-elective Patients (n=5261)** | **4145** | **1116** |  | |
| HFRS (non-frail; n=3494 [66.4%]) | 2847 | 647 | 0.12  (0.09-0.15; p<0.001) | 0.08  (0.06-0.11; p<0.001) |
| HFRS (frail; n=1767 [33.6%]) | 1298 | 469 |  |  |
| **Patients alive at discharge (n=6359)** | **5245** | **1114** |  | |
| HFRS (non-frail; n=4779 [75.2%]) | 4066 | 713 | 0.12  (0.09-0.14; p<0.001) | 0.12  (0.07-0.14; p<0.001) |
| HFRS (frail; n=1580 [24.8%]) | 1179 | 401 |  |  |
| HFRS – hospital frailty risk score; CFS – Clinical Frailty Scale  ^(1)^ Spearman correlation based on continuous variables  ^(2)^ Kappa agreement based on dichotomous variables | | | | |

**Supplementary Table 6:** Subgroup analysis: Unadjusted and adjusted Cox proportional hazards regression for CFS and the HFRS as continuous variables, adjusting for ANZROD, and sex CFS and HFRS or (a) Patients ≥65 years of age, (b) Patients ≥75 years of age, (c) Patients needing mechanical ventilation, (d) elective patients, (e) non-elective patients and (f) patients who survived to hospital discharge.

|  | **Unadjusted** | **Adjusted** |
| --- | --- | --- |
|  | **HR (95%-CI)** | **HR (95%-CI)** |
| **Patients ≥75 years (n=1683)** | | |
| **CFS** | 1.32 (1.23-1.42) | **1.18** (1.10-1.27) |
| **HFRS^**^** | 1.35 (1.23-1.47) | 1.05 (0.95-1.16) |
| **Patients needing mechanical ventilation^*^ (n=3266)** | | |
| **CFS** | 1.25 (1.19-1.32) | **1.14** (1.08-1.20) |
| **HFRS^**^** | 1.30 (1.21-1.39) | **1.17** (1.08-1.28) |
| **Elective Patients (n=1740)** | | |
| **CFS** | 1.44 (1.19-1.73) | **1.31** (1.08-1.58) |
| **HFRS^**^** | 2.06 (1.30-3.26) | 1.44 (0.86-2.41) |
| **Non-elective Patients (n=5261)** | | |
| **CFS** | 1.39 (1.34-1.44) | **1.25** (1.20-1.30) |
| **HFRS^**^** | 1.21 (1.14-1.28) | 1.02 (0.96-1.09) |
| **Patients alive at discharge (n=6359)** | | |
| **CFS** | 1.61 (1.52-1.72) | **1.51** (1.42-1.61) |
| **HFRS^**^** | 1.33 (1.21-1.45) | **1.13** (1.02-1.25) |
| ^*^Adjusted for sex  ^**^5-unit increase HFRS  Except for Adjusted HFRS for patients ≥65 years (p=0.27) and Adjusted HFRS for patients needing mechanical ventilation (p=0.94), all p-values were <0.001.  CFS - Clinical frailty score; HFRS - hospital frailty risk score; HR - Hazard ratio | | |

**Supplementary Table 7:** Logistic Regression analysis for short-term and long-term mortalities. Unadjusted and adjusted for sex and ANZROD. The odds ratio (OR) in bold represent statistically significant results. Graphically presented in Figure 2.

| **Frail - outcome** | **CFS** | | | | **HFRS** | | | |
| --- | --- | --- | --- | --- | --- | --- | --- | --- |
|  | **Unadjusted** | | **Adjusted*** | | **Unadjusted** | | **Adjusted*** | |
|  | **OR** | **95%-CI** | **OR** | **95%-CI** | **OR** | **95%-CI** | **OR** | **95%-CI** |
| **All patients (n=7001)** | | | | | | | | |
| ICU mortality | 1.29 | 1.21-1.37 | **1.10** | 1.02-1.18 | 1.38 | 1.27-1.50 | 0.99 | 0.88-1.12 |
| Hospital mortality | 1.36 | 1.29-1.44 | **1.21** | 1.13-1.29 | 1.46 | 1.36-1.58 | **1.11** | 1.00-1.22 |
| 28-day mortality | 1.38 | 1.31-1.45 | **1.23** | 1.15-1.31 | 1.45 | 1.35-1.56 | 1.09 | 0.98-1.21 |
| 90-day mortality | 1.45 | 1.38-1.52 | **1.33** | 1.25-1.41 | 1.48 | 1.38-1.59 | **1.14** | 1.05-1.25 |
| 6-month mortality | 1.49 | 1.42-1.56 | **1.38** | 1.31-1.46 | 1.48 | 1.38-1.58 | **1.14** | 1.04-1.24 |
| 12-month mortality | 1.49 | 1.43-1.56 | **1.38** | 1.31-1.46 | 1.47 | 1.38-1.57 | **1.13** | 1.04-1.23 |
| **Patients ≥75 years (n=1683)** | | | | | | | | |
| ICU mortality | 1.12 | 0.99-1.27 | 0.94 | 0.81-1.08 | 1.17 | 0.99-1.39 | 0.81 | 0.64-1.02 |
| Hospital mortality | 1.25 | 1.13-1.38 | 1.09 | 0.97-1.23 | 1.32 | 1.16-1.51 | 1.01 | 0.85-1.21 |
| 28-day mortality | 1.24 | 1.12-1.37 | 1.07 | 0.94-1.20 | 1.33 | 1.17-1.52 | 1.01 | 0.85-1.21 |
| 90-day mortality | 1.34 | 1.23-1.47 | **1.20** | 1.08-1.34 | 1.49 | 1.32-1.68 | **1.21** | 1.04-1.40 |
| 6-month mortality | 1.35 | 1.24-1.47 | **1.22** | 1.10-1.35 | 1.47 | 1.31-1.65 | **1.18** | 1.02-1.36 |
| 12-month mortality | 1.36 | 1.26-1.48 | **1.24** | 1.12-1.37 | 1.47 | 1.31-1.64 | **1.17** | 1.02-1.35 |
| **Patients needing mechanical ventilation^**^ (n=3266)** | | | | | | | | |
| ICU mortality | 1.19 | 1.11-1.28 | **1.10** | 1.00-1.20 | 1.34 | 1.22-1.48 | 1.00 | 0.87-1.14 |
| Hospital mortality | 1.25 | 1.17-1.33 | **1.19** | 1.10-1.29 | 1.42 | 1.29-1.55 | 1.09 | 0.97-1.23 |
| 28-day mortality | 1.24 | 1.16-1.32 | **1.17** | 1.08-1.28 | 1.38 | 1.26-1.51 | 1.05 | 0.92-1.18 |
| 90-day mortality | 1.27 | 1.19-1.35 | **1.23** | 1.13-1.33 | 1.40 | 1.28-1.53 | 1.06 | 0.95-1.19 |
| 6-month mortality | 1.27 | 1.20-1.35 | **1.23** | 1.14-1.33 | 1.40 | 1.28-1.52 | 1.01 | 0.95-1.18 |
| 12-month mortality | 1.29 | 1.21-1.37 | **1.26** | 1.17-1.35 | 1.38 | 1.27-1.50 | 1.01 | 0.93-1.16 |
| **Elective Patients (n=1740)** | | | | | | | | |
| ICU mortality | 1.36 | 0.91-2.05 | 1.16 | 0.75-1.81 | 1.89 | 0.69-5.15 | 0.95 | 0.24-3.80 |
| Hospital mortality | 1.38 | 1.03-1.87 | 1.21 | 0.88-1.68 | 2.50 | 1.33-4.73 | 1.64 | 0.76-3.55 |
| 28-day mortality | 1.35 | 1.00-1.82 | 1.19 | 0.86-1.65 | 2.43 | 1.27-4.64 | 1.59 | 0.73-3.50 |
| 90-day mortality | 1.41 | 1.12-1.79 | 1.26 | 0.97-1.62 | 2.67 | 1.61-4.44 | **1.86** | 1.03-3.35 |
| 6-month mortality | 1.48 | 1.20-1.82 | **1.34** | 1.07-1.67 | 2.40 | 1.48-3.90 | 1.67 | 0.96-2.93 |
| 12-month mortality | 1.46 | 1.21-1.78 | **1.31** | 1.06-1.61 | 2.04 | 1.25-3.32 | 1.32 | 0.74-2.35 |
| **Non-elective Patients (n=5261)** | | | | | | | | |
| ICU mortality | 1.25 | 1.18-1.33 | **1.09** | 1.01-1.17 | 1.18 | 1.07-1.29 | 0.93 | 0.83-1.05 |
| Hospital mortality | 1.32 | 1.26-1.39 | **1.19** | 1.12-1.27 | 1.25 | 1.16-1.36 | 1.04 | 0.94-1.15 |
| 28-day mortality | 1.34 | 1.27-1.41 | **1.22** | 1.14-1.30 | 1.24 | 1.15-1.35 | 1.02 | 0.92-1.14 |
| 90-day mortality | 1.41 | 1.35-1.48 | **1.31** | 1.24-1.39 | 1.27 | 1.18-1.36 | 1.06 | 0.97-1.17 |
| 6-month mortality | 1.45 | 1.38-1.52 | **1.36** | 1.29-1.44 | 1.26 | 1.18-1.36 | 1.06 | 0.97-1.15 |
| 12-month mortality | 1.46 | 1.39-1.52 | **1.37** | 1.30-1.45 | 1.26 | 1.18-1.35 | 1.06 | 0.97-1.15 |
| **Patients alive at discharge (n=6359)** | | | | | | | | |
| ICU mortality | - | - | - | - | - | - | - | - |
| Hospital mortality | - | - | - | - | - | - | - | - |
| 28-day mortality | 1.80 | 1.43-2.27 | **1.63** | 1.28-2.08 | 1.27 | 0.88-1.82 | 0.95 | 0.62-1.46 |
| 90-day mortality | 1.67 | 1.53-1.83 | **1.55** | 1.41-1.70 | 1.43 | 1.26-1.63 | **1.21** | 1.04-1.40 |
| 6-month mortality | 1.67 | 1.55-1.80 | **1.56** | 1.45-1.69 | 1.41 | 1.27-1.57 | **1.17** | 1.04-1.32 |
| 12-month mortality | 1.64 | 1.53-1.76 | **1.54** | 1.43-1.65 | 1.39 | 1.26-1.53 | **1.16** | 1.03-1.30 |
| ^*^Adjusted for male sex and ANZROD  ^**^ Adjusted for ANZROD  CFS - Clinical frailty score, HFRS - hospital frailty risk score | | | | | | | | |

**Supplementary Table 8:** Proportion of patients without a documented CFS in the ANZICS database. reduced with time.

| **Month/year** | **20,457 hospitalisations during the study period** | | | **14,943 patients that were linked** | | |
| --- | --- | --- | --- | --- | --- | --- |
|  | **CFS recorded** | **CFS missing** | **Missing (%)** | **CFS recorded** | **CFS missing** | **Missing (%)** |
| April 2017 | 229 | 1169 | 83.6% | 189 | 899 | 82.6% |
| May 2017 | 244 | 1256 | 83.7% | 202 | 934 | 82.2% |
| June 2017 | 280 | 1193 | 80.9% | 236 | 844 | 78.1% |
| July 2017 | 325 | 1125 | 77.6% | 256 | 807 | 75.9% |
| August 2017 | 588 | 1002 | 63.0% | 441 | 739 | 62.6% |
| September 2017 | 510 | 900 | 63.8% | 378 | 642 | 62.9% |
| October 2017 | 522 | 874 | 62.6% | 407 | 626 | 60.6% |
| November 2017 | 556 | 676 | 54.9% | 434 | 496 | 53.3% |
| December 2017 | 729 | 507 | 41.0% | 563 | 388 | 40.8% |
| January 2018 | 761 | 464 | 37.9% | 593 | 312 | 34.5% |
| February 2018 | 755 | 474 | 38.6% | 611 | 340 | 35.8% |
| March 2018 | 936 | 486 | 34.2% | 679 | 347 | 33.8% |
| April 2018 | 892 | 321 | 26.5% | 654 | 234 | 26.4% |
| May 2018 | 1066 | 315 | 22.8% | 798 | 222 | 21.8% |
| June 2018 | 1046 | 256 | 19.7% | 560 | 113 | 16.8% |

**Supplementary Figure 1:** Prevalence of frailty measure by CFS and HFRS across different age categories.

**Supplementary Figure 2:** Kaplan Meier curves between frail and non-frail patients (treated as a dichotomous variable) for CFS and HFRS.

**
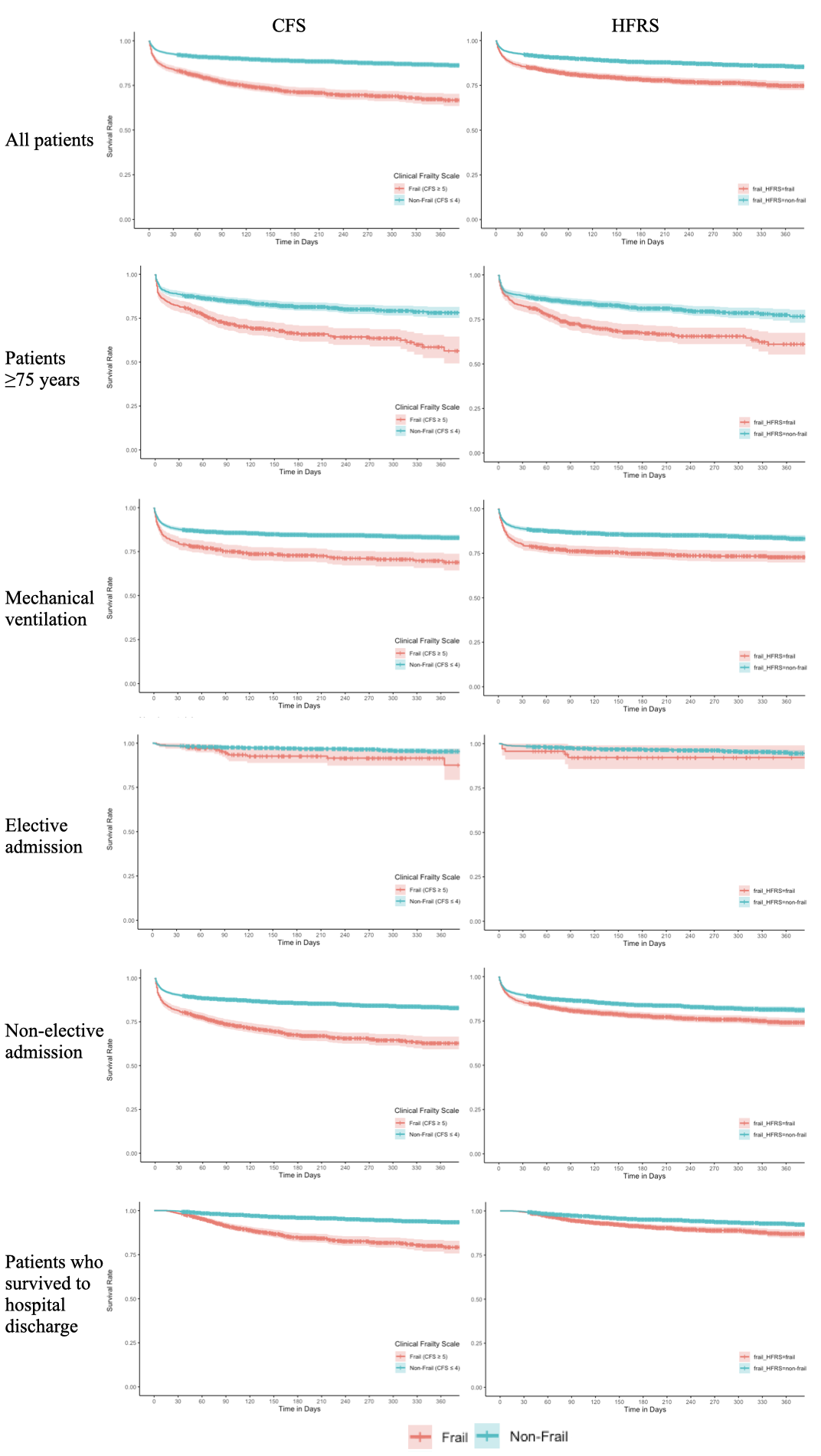
**

**Supplementary Figure 3:** Area under the receiver operator curve for short-term mortality. The CFS was significantly better than the HFRS. However, CFS and HFRS must both be measuring different aspects, because the combination of the two was better than either measure on its own. The bottom 2 graphs showed that while both variables may be predictive of mortality, neither provided any worthwhile improvement on top of ANZROD.


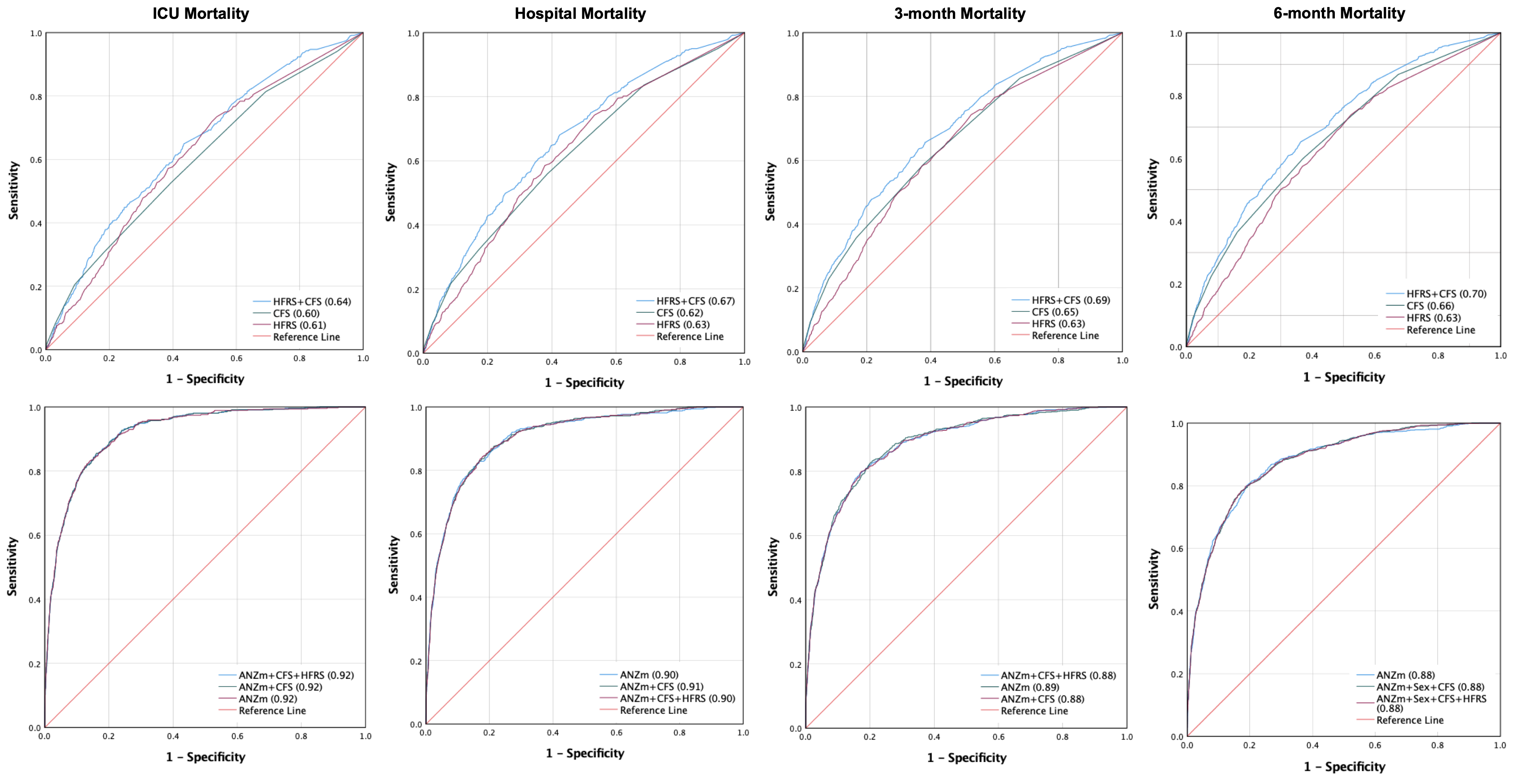


ANZm – ANZROD + male sex, CFS clinical frailty scale, HFRS – hospital frailty risk score

**Supplementary Figure 4:** Area under the receiver operator curve for 1-year mortality for patients ≥75 years of age and those needing mechanical ventilation.


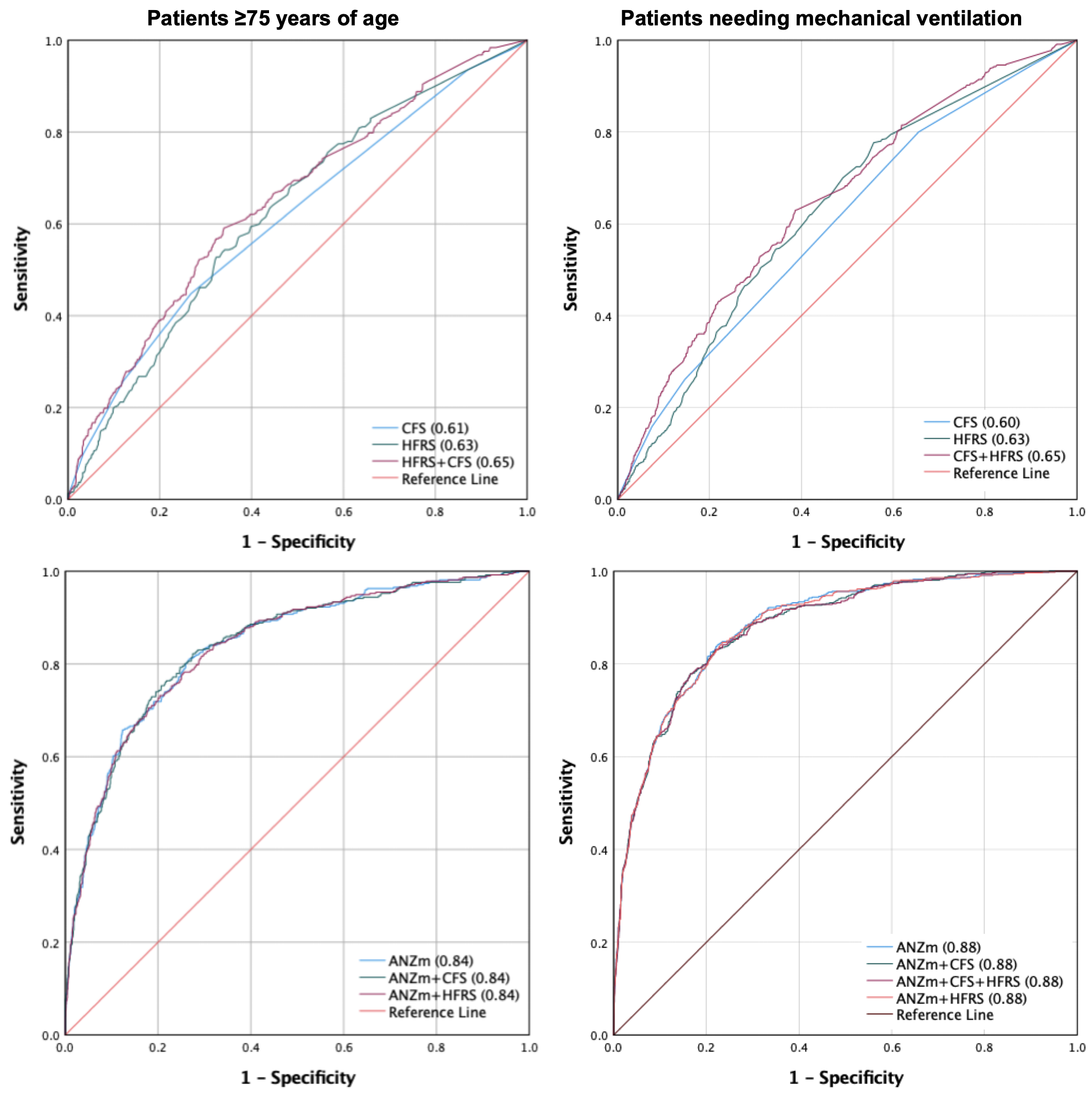


ANZm – ANZROD + male sex, CFS clinical frailty scale, HFRS – hospital frailty risk score
